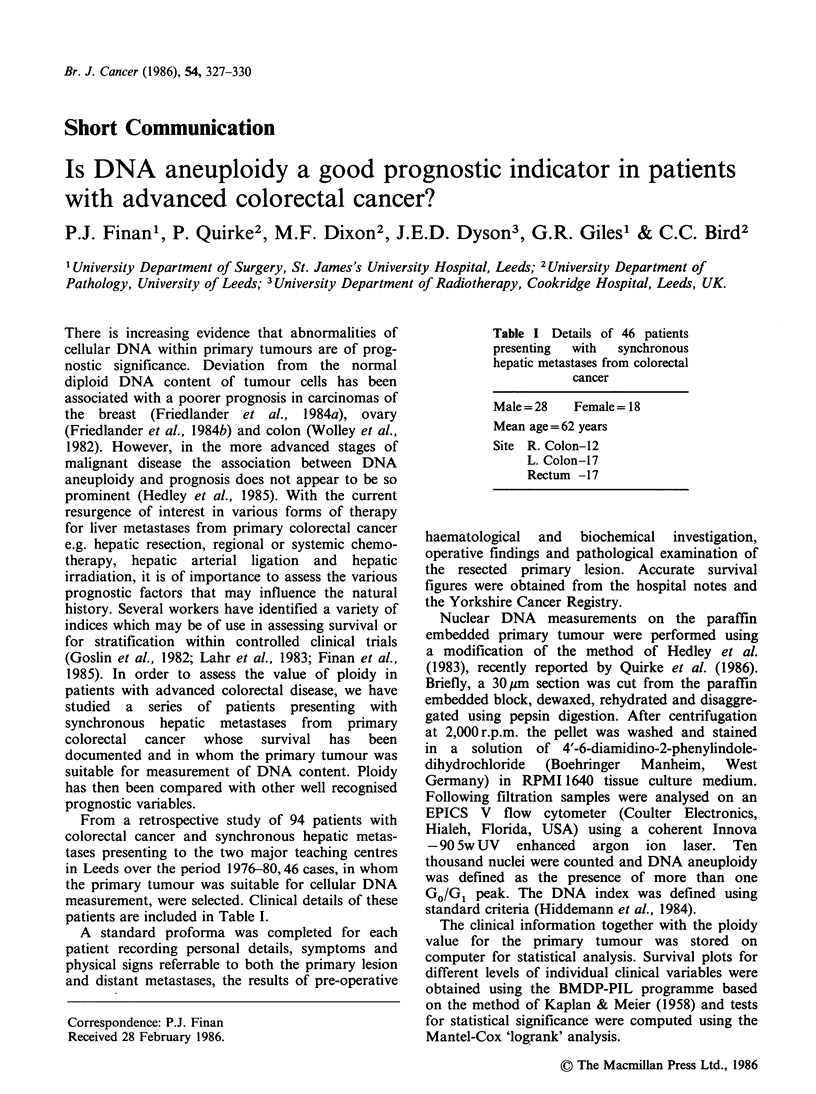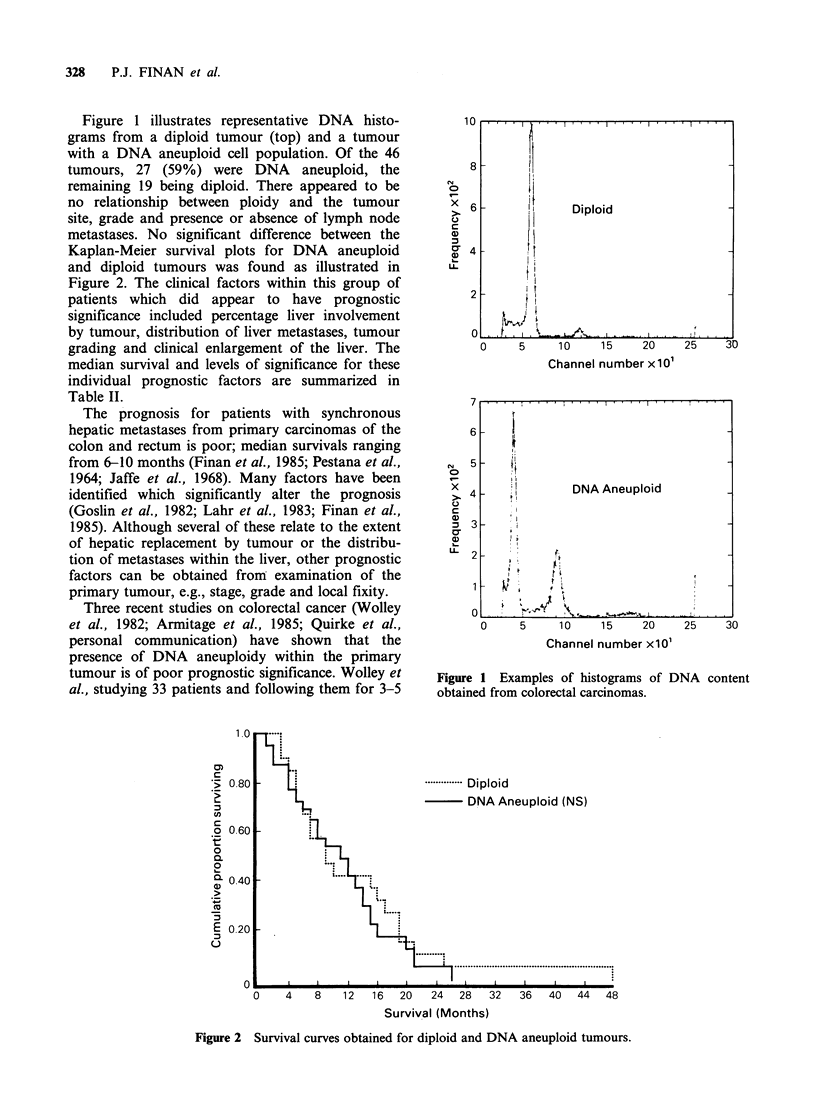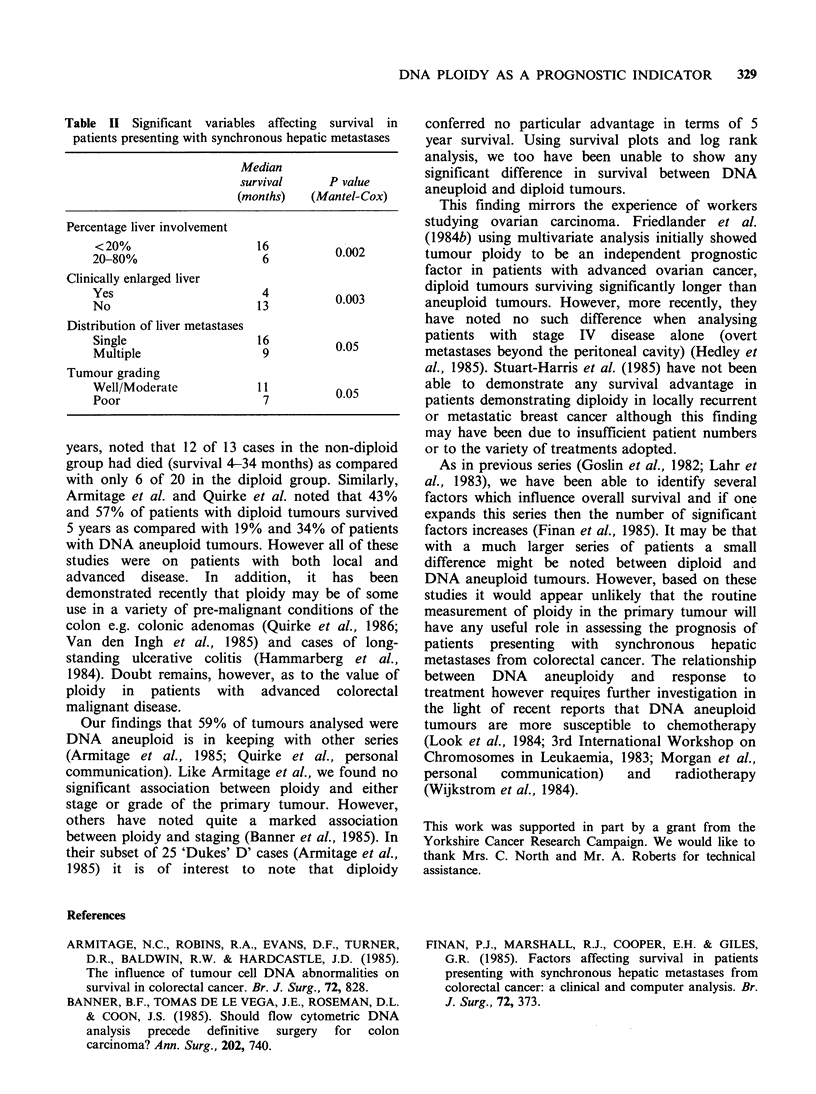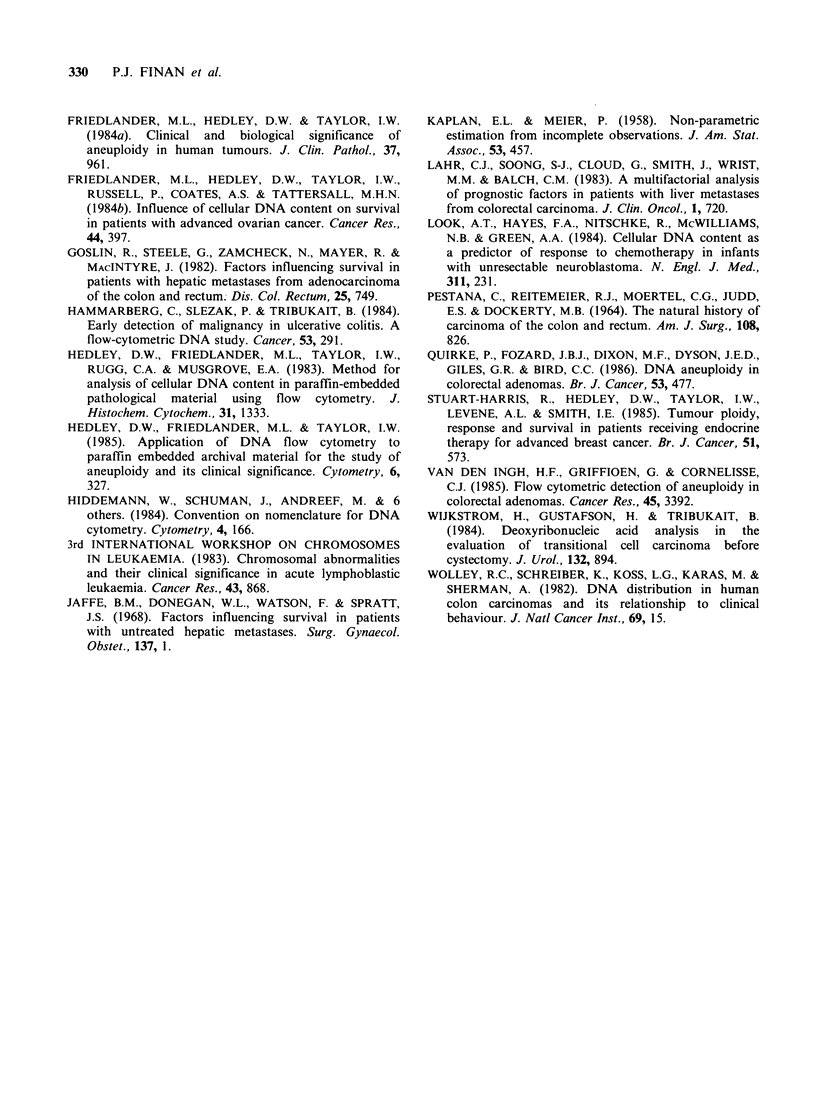# Is DNA aneuploidy a good prognostic indicator in patients with advanced colorectal cancer?

**DOI:** 10.1038/bjc.1986.180

**Published:** 1986-08

**Authors:** P. J. Finan, P. Quirke, M. F. Dixon, J. E. Dyson, G. R. Giles, C. C. Bird


*Br. J. Cancer* (1986), **54**, 327–330

### Short Communication

### Is DNA aneuploidy a good prognostic indicator in patients with advanced colorectal cancer?

P.J. Finan[1], P. Quirke[2], M.F. Dixon[2], J.E.D. Dyson[3], G.R. Giles[1] & C.C. Bird[2]

[1]*University Department of Surgery, St. James's University Hospital, Leeds;* [2]*University Department of Pathology, University of Leeds;* [3]*University Department of Radiotherapy, Cookridge Hospital, Leeds, UK.*

There is increasing evidence that abnormalities of cellular DNA within primary tumours are of prognostic significance. Deviation from the normal diploid DNA content of tumour cells has been associated with a poorer prognosis in carcinomas of the breast (Friedlander *et al.*, 1984*a*), ovary (Friedlander *et al.*, 1984*b*) and colon (Wolley *et al.*, 1982). However, in the more advanced stages of malignant disease the association between DNA aneuploidy and prognosis does not appear to be so prominent (Hedley *et al.*, 1985). With the current resurgence of interest in various forms of therapy for liver metastases from primary colorectal cancer e.g. hepatic resection, regional or systemic chemotherapy, hepatic arterial ligation and hepatic irradiation, it is of importance to assess the various prognostic factors that may influence the natural history. Several workers have identified a variety of indices which may be of use in assessing survival or for stratification within controlled clinical trials (Goslin *et al.*, 1982; Lahr *et al.*, 1983; Finan *et al.*, 1985). In order to assess the value of ploidy in patients with advanced colorectal disease, we have studied a series of patients presenting with synchronous hepatic metastases from primary colorectal cancer whose survival has been documented and in whom the primary tumour was suitable for measurement of DNA content. Ploidy has then been compared with other well recognised prognostic variables.

From a retrospective study of 94 patients with colorectal cancer and synchronous hepatic metastases presenting to the two major teaching centres in Leeds over the period 1976–80, 46 cases, in whom the primary tumour was suitable for cellular DNA measurement, were selected. Clinical details of these patients are included in Table I.

A standard proforma was completed for each patient recording personal details, symptoms and physical signs referrable to both the primary lesion and distant metastases, the results of pre-operative haematological and biochemical investigation, operative findings and pathological examination of the resected primary lesion. Accurate survival figures were obtained from the hospital notes and the Yorkshire Cancer Registry.

**Table I** Details of 46 patients presenting with synchronous hepatic metastases from colorectal cancer

| | | |
|---|---|---|
| Male = 28 | | Female = 18 |
| Mean age = 62 years | | |
| Site | R. Colon–12 | |
| | L. Colon–17 | |
| | Rectum –17 | |

Nuclear DNA measurements on the paraffin embedded primary tumour were performed using a modification of the method of Hedley *et al.* (1983), recently reported by Quirke *et al.* (1986). Briefly, a 30 $\mu$m section was cut from the paraffin embedded block, dewaxed, rehydrated and disaggregated using pepsin digestion. After centrifugation at 2,000 r.p.m. the pellet was washed and stained in a solution of 4′-6-diamidino-2-phenylindole-dihydrochloride (Boehringer Manheim, West Germany) in RPMI 1640 tissue culture medium. Following filtration samples were analysed on an EPICS V flow cytometer (Coulter Electronics, Hialeh, Florida, USA) using a coherent Innova −90 5w UV enhanced argon ion laser. Ten thousand nuclei were counted and DNA aneuploidy was defined as the presence of more than one $G_0/G_1$ peak. The DNA index was defined using standard criteria (Hiddemann *et al.*, 1984).

The clinical information together with the ploidy value for the primary tumour was stored on computer for statistical analysis. Survival plots for different levels of individual clinical variables were obtained using the BMDP-PIL programme based on the method of Kaplan & Meier (1958) and tests for statistical significance were computed using the Mantel-Cox 'logrank' analysis.

Correspondence: P.J. Finan
Received 28 February 1986.

© The Macmillan Press Ltd., 1986

Figure 1 illustrates representative DNA histograms from a diploid tumour (top) and a tumour with a DNA aneuploid cell population. Of the 46 tumours, 27 (59%) were DNA aneuploid, the remaining 19 being diploid. There appeared to be no relationship between ploidy and the tumour site, grade and presence or absence of lymph node metastases. No significant difference between the Kaplan-Meier survival plots for DNA aneuploid and diploid tumours was found as illustrated in Figure 2. The clinical factors within this group of patients which did appear to have prognostic significance included percentage liver involvement by tumour, distribution of liver metastases, tumour grading and clinical enlargement of the liver. The median survival and levels of significance for these individual prognostic factors are summarized in Table II.

The prognosis for patients with synchronous hepatic metastases from primary carcinomas of the colon and rectum is poor; median survivals ranging from 6–10 months (Finan *et al.*, 1985; Pestana *et al.*, 1964; Jaffe *et al.*, 1968). Many factors have been identified which significantly alter the prognosis (Goslin *et al.*, 1982; Lahr *et al.*, 1983; Finan *et al.*, 1985). Although several of these relate to the extent of hepatic replacement by tumour or the distribution of metastases within the liver, other prognostic factors can be obtained from examination of the primary tumour, e.g., stage, grade and local fixity.

Three recent studies on colorectal cancer (Wolley *et al.*, 1982; Armitage *et al.*, 1985; Quirke *et al.*, personal communication) have shown that the presence of DNA aneuploidy within the primary tumour is of poor prognostic significance. Wolley *et al.*, studying 33 patients and following them for 3–5

**Figure 1** Examples of histograms of DNA content obtained from colorectal carcinomas.

**Figure 2** Survival curves obtained for diploid and DNA aneuploid tumours.

**Table II** Significant variables affecting survival in patients presenting with synchronous hepatic metastases

| | *Median survival (months)* | *P value (Mantel-Cox)* |
|---|---|---|
| Percentage liver involvement | | |
| <20% | 16 | 0.002 |
| 20–80% | 6 | |
| Clinically enlarged liver | | |
| Yes | 4 | 0.003 |
| No | 13 | |
| Distribution of liver metastases | | |
| Single | 16 | 0.05 |
| Multiple | 9 | |
| Tumour grading | | |
| Well/Moderate | 11 | 0.05 |
| Poor | 7 | |

years, noted that 12 of 13 cases in the non-diploid group had died (survival 4–34 months) as compared with only 6 of 20 in the diploid group. Similarly, Armitage *et al.* and Quirke *et al.* noted that 43% and 57% of patients with diploid tumours survived 5 years as compared with 19% and 34% of patients with DNA aneuploid tumours. However all of these studies were on patients with both local and advanced disease. In addition, it has been demonstrated recently that ploidy may be of some use in a variety of pre-malignant conditions of the colon e.g. colonic adenomas (Quirke *et al.*, 1986; Van den Ingh *et al.*, 1985) and cases of long-standing ulcerative colitis (Hammarberg *et al.*, 1984). Doubt remains, however, as to the value of ploidy in patients with advanced colorectal malignant disease.

Our findings that 59% of tumours analysed were DNA aneuploid is in keeping with other series (Armitage *et al.*, 1985; Quirke *et al.*, personal communication). Like Armitage *et al.*, we found no significant association between ploidy and either stage or grade of the primary tumour. However, others have noted quite a marked association between ploidy and staging (Banner *et al.*, 1985). In their subset of 25 'Dukes' D' cases (Armitage *et al.*, 1985) it is of interest to note that diploidy conferred no particular advantage in terms of 5 year survival. Using survival plots and log rank analysis, we too have been unable to show any significant difference in survival between DNA aneuploid and diploid tumours.

This finding mirrors the experience of workers studying ovarian carcinoma. Friedlander *et al.* (1984*b*) using multivariate analysis initially showed tumour ploidy to be an independent prognostic factor in patients with advanced ovarian cancer, diploid tumours surviving significantly longer than aneuploid tumours. However, more recently, they have noted no such difference when analysing patients with stage IV disease alone (overt metastases beyond the peritoneal cavity) (Hedley *et al.*, 1985). Stuart-Harris *et al.* (1985) have not been able to demonstrate any survival advantage in patients demonstrating diploidy in locally recurrent or metastatic breast cancer although this finding may have been due to insufficient patient numbers or to the variety of treatments adopted.

As in previous series (Goslin *et al.*, 1982; Lahr *et al.*, 1983), we have been able to identify several factors which influence overall survival and if one expands this series then the number of significant factors increases (Finan *et al.*, 1985). It may be that with a much larger series of patients a small difference might be noted between diploid and DNA aneuploid tumours. However, based on these studies it would appear unlikely that the routine measurement of ploidy in the primary tumour will have any useful role in assessing the prognosis of patients presenting with synchronous hepatic metastases from colorectal cancer. The relationship between DNA aneuploidy and response to treatment however requires further investigation in the light of recent reports that DNA aneuploid tumours are more susceptible to chemotherapy (Look *et al.*, 1984; 3rd International Workshop on Chromosomes in Leukaemia, 1983; Morgan *et al.*, personal communication) and radiotherapy (Wijkstrom *et al.*, 1984).

This work was supported in part by a grant from the Yorkshire Cancer Research Campaign. We would like to thank Mrs. C. North and Mr. A. Roberts for technical assistance.

**References**

ARMITAGE, N.C., ROBINS, R.A., EVANS, D.F., TURNER, D.R., BALDWIN, R.W. & HARDCASTLE, J.D. (1985). The influence of tumour cell DNA abnormalities on survival in colorectal cancer. *Br. J. Surg.*, **72**, 828.

BANNER, B.F., TOMAS DE LE VEGA, J.E., ROSEMAN, D.L. & COON, J.S. (1985). Should flow cytometric DNA analysis precede definitive surgery for colon carcinoma? *Ann. Surg.*, **202**, 740.

FINAN, P.J., MARSHALL, R.J., COOPER, E.H. & GILES, G.R. (1985). Factors affecting survival in patients presenting with synchronous hepatic metastases from colorectal cancer: a clinical and computer analysis. *Br. J. Surg.*, **72**, 373.

FRIEDLANDER, M.L., HEDLEY, D.W. & TAYLOR, I.W. (1984*a*). Clinical and biological significance of aneuploidy in human tumours. *J. Clin. Pathol.*, **37**, 961.

FRIEDLANDER, M.L., HEDLEY, D.W., TAYLOR, I.W., RUSSELL, P., COATES, A.S. & TATTERSALL, M.H.N. (1984*b*). Influence of cellular DNA content on survival in patients with advanced ovarian cancer. *Cancer Res.*, **44**, 397.

GOSLIN, R., STEELE, G., ZAMCHECK, N., MAYER, R. & MACINTYRE, J. (1982). Factors influencing survival in patients with hepatic metastases from adenocarcinoma of the colon and rectum. *Dis. Col. Rectum*, **25**, 749.

HAMMARBERG, C., SLEZAK, P. & TRIBUKAIT, B. (1984). Early detection of malignancy in ulcerative colitis. A flow-cytometric DNA study. *Cancer*, **53**, 291.

HEDLEY, D.W., FRIEDLANDER, M.L., TAYLOR, I.W., RUGG, C.A. & MUSGROVE, E.A. (1983). Method for analysis of cellular DNA content in paraffin-embedded pathological material using flow cytometry. *J. Histochem. Cytochem.*, **31**, 1333.

HEDLEY, D.W., FRIEDLANDER, M.L. & TAYLOR, I.W. (1985). Application of DNA flow cytometry to paraffin embedded archival material for the study of aneuploidy and its clinical significance. *Cytometry*, **6**, 327.

HIDDEMANN, W., SCHUMAN, J., ANDREEF, M. & 6 others. (1984). Convention on nomenclature for DNA cytometry. *Cytometry*, **4**, 166.

3rd INTERNATIONAL WORKSHOP ON CHROMOSOMES IN LEUKAEMIA. (1983). Chromosomal abnormalities and their clinical significance in acute lymphoblastic leukaemia. *Cancer Res.*, **43**, 868.

JAFFE, B.M., DONEGAN, W.L., WATSON, F. & SPRATT, J.S. (1968). Factors influencing survival in patients with untreated hepatic metastases. *Surg. Gynaecol. Obstet.*, **137**, 1.

KAPLAN, E.L. & MEIER, P. (1958). Non-parametric estimation from incomplete observations. *J. Am. Stat. Assoc.*, **53**, 457.

LAHR, C.J., SOONG, S-J., CLOUD, G., SMITH, J., WRIST, M.M. & BALCH, C.M. (1983). A multifactorial analysis of prognostic factors in patients with liver metastases from colorectal carcinoma. *J. Clin. Oncol.*, **1**, 720.

LOOK, A.T., HAYES, F.A., NITSCHKE, R., MCWILLIAMS, N.B. & GREEN, A.A. (1984). Cellular DNA content as a predictor of response to chemotherapy in infants with unresectable neuroblastoma. *N. Engl. J. Med.*, **311**, 231.

PESTANA, C., REITEMEIER, R.J., MOERTEL, C.G., JUDD, E.S. & DOCKERTY, M.B. (1964). The natural history of carcinoma of the colon and rectum. *Am. J. Surg.*, **108**, 826.

QUIRKE, P., FOZARD, J.B.J., DIXON, M.F., DYSON, J.E.D., GILES, G.R. & BIRD, C.C. (1986). DNA aneuploidy in colorectal adenomas. *Br. J. Cancer*, **53**, 477.

STUART-HARRIS, R., HEDLEY, D.W., TAYLOR, I.W., LEVENE, A.L. & SMITH, I.E. (1985). Tumour ploidy, response and survival in patients receiving endocrine therapy for advanced breast cancer. *Br. J. Cancer*, **51**, 573.

VAN DEN INGH, H.F., GRIFFIOEN, G. & CORNELISSE, C.J. (1985). Flow cytometric detection of aneuploidy in colorectal adenomas. *Cancer Res.*, **45**, 3392.

WIJKSTROM, H., GUSTAFSON, H. & TRIBUKAIT, B. (1984). Deoxyribonucleic acid analysis in the evaluation of transitional cell carcinoma before cystectomy. *J. Urol.*, **132**, 894.

WOLLEY, R.C., SCHREIBER, K., KOSS, L.G., KARAS, M. & SHERMAN, A. (1982). DNA distribution in human colon carcinomas and its relationship to clinical behaviour. *J. Natl Cancer Inst.*, **69**, 15.